# Editorial: Multi-omics approaches and translational medicine in T cell dysregulation in autoimmunity, cancer, and chronic infections

**DOI:** 10.3389/fimmu.2023.1279417

**Published:** 2023-09-15

**Authors:** Haijing Wu, Ling Lu

**Affiliations:** ^1^ Department of Dermatology, The Second Xiangya Hospital of Central South University, Hunan Key Laboratory of Medical Epigenomics, Changsha, Hunan, China; ^2^ Hepatobiliary Center, The First Affiliated Hospital of Nanjing Medical University and Research Unit of Liver Transplantation and Transplant Immunology, Chinese Academy of Medical Sciences, Nanjing, China

**Keywords:** genome-wide association studies, epigenetic modifications, single-cell transcriptome sequencing, autoimmunity, chronic infection, cancer, T cells

T cells play a crucial role in the host defense against pathogens and maintain tolerance with normal microbiota in the body. However, sometimes exogenous triggers may disrupt T cell homeostasis, which breaks down T cell tolerance and induces T cell exhaustion leading to autoimmunity, chronic infections, and cancers. Recent research advances in immunology have introduced new technologies to investigate T cell regulation at single cell level and on omics scale, including genome-wide association studies, epigenomes (DNA methylation, histone modification, and microRNA) profile, and transcriptome in T cells. The integration of these information will provide new insights on the mechanisms of the T cell dysregulation under pathogenic conditions. Regarding this Research Topic is very attractive and promising, which may help to design novel therapeutics or develop biomarkers for prediction, diagnosis, prognosis, and treatment response.

Due to the development of new technologies, single cell sequencing is applied to explore the new functions of T cells in various diseases. In this Research Topic, Han et al. applied high-throughput single-cell RNA sequencing technology and reported functional heterogeneity among the hepatic CD8^+^ T cells subsets in dnTGFβRII mice. CD8^+^ T cells were confirmed the key cells leading to the pathogenesis of primary biliary cholangitis (PBC) in dnTGFβRII mice and identified the terminally differentiated CD8αα T cells and CD8αβ T cell subsets in the liver of dnTGFβRII mice.

In the field of epigenetic studies, Kong et al. reported β-Glucuronidase (GUSB) facilitated proliferation, invasion, as well as migration of human hepatocellular carcinoma cells and downregulated PD-L1 expression by promoting miR-513a-5p. Peng et al. proposed that Circular RNA circNUP214 is an abundant and stable circRNA in rheumatoid arthritis (RA) patients that can potentially differentiate RA patients from healthy subjects. Qing et al. described that 64 shared differentially expressed genes (SDEGs) and 28 SIRGs were identified in patients with IgA nephropathy (IgAN) and inflammatory bowel disease (IBD), and the area under the receiver operating characteristic curve (ROC) of 64 SDEGs was calculated and two genes (MVP, PDXK) with high area under the curve (AUC) in both IgAN and IBD were screened out as potential diagnostic biomarkers.

Besides 5 original articles, 2 reviews on T cell senescence and regeneration also provided interesting comprehensive information for further understanding of T cell functions in diseases.

The goal of this Research Topic is to explore more updated information of functions of T cells by new omics methodologies. This Research Topic aims to highlight the most advanced achievements in the genome-wide association studies, epigenomes (DNA methylation, histone modification, and microRNA) profile, and transcriptome in T cell, which may help design novel therapeutics or develop biomarkers for prediction, diagnosis, prognosis, and treatment response.

## Author contributions

HW: Writing – original draft. LL: Writing – review & editing.

